# Systems all the way down: embracing complexity in mental health research

**DOI:** 10.1186/s12916-020-01668-w

**Published:** 2020-07-14

**Authors:** Eiko I. Fried, Donald J. Robinaugh

**Affiliations:** 1 Department of Clinical Psychology, Leiden University, Leiden, The Netherlands; 2 Department of Psychiatry, Harvard Medical School & Massachusetts General Hospital, Boston, MA USA

## Abstract

In this editorial for the collection on complexity in mental health research, we introduce and summarize the inaugural contributions to this collection: a series of theoretical, methodological, and empirical papers that aim to chart a path forward for investigating mental health in all its complexity. A central theme emerges from these contributions: if we are to make genuine progress in explaining, predicting, and treating mental illness, we must study the *systems* from which psychopathology emerges. As the articles in this collection make clear, the systems that give rise to psychopathology encompass a host of components across biological, psychological, and social levels of analysis, intertwined in a web of complex interactions. The task of advancing our understanding of these systems will be a challenging one. Yet, this challenge presents a unique opportunity. From physics to ecology, there is a rapidly evolving body of interdisciplinary research dedicated to investigating complex systems. This work provides clear guidance for psychiatric research, opportunities for collaboration, and a set of tools and concepts from which we can draw in our efforts to understand mental health, helping us move toward our ultimate aim of improving the prevention and treatment of psychopathology.

## Background

“The crises we face are systemic in nature. To overcome those crises we need to understand how systems work. To arrive at such an understanding we need to think systemically.” [1]

Throughout the twentieth century, much of psychiatry aspired to reductionist simplicity. Whether in the Freudian unconscious, the human genome, or dysfunctional neurobiology, researchers sought to identify *the* underlying cause of the troubles faced by their patients. However, far from uncovering simple etiologies, the past century of psychiatric research has revealed systematic complexity, leading to a growing recognition that mental disorders are dauntingly complex phenomena [2, 3]. Yet, despite this growing acceptance, there has been little change in how we study psychopathology, and much of our research remains stubbornly rooted in the monocausal framework. If we are to make genuine progress in explaining, predicting, and treating mental illness, it is critical that we embrace the complexity inherent in these disorders in our theories, methods, and empirical research.

In this collection, we present a series of papers that take up this charge. From these contributions, a central theme emerges: mental disorders arise from a host of components across biological, psychological, and social levels of analysis, intertwined in a web of complex interactions. To understand mental disorders, it is not sufficient to understand single components alone: we cannot hope to understand depression from studying only the amygdala any more than we can understand the weather by careful examination of water droplets. We must go further and understand both the individual components *and* the complex interactions among them. That is, we should aim to understand the *systems* from which psychopathology emerges.

Achieving such understanding will be no easy task. The systems giving rise to mental illness are massively multifactorial, and there is little evidence that any one factor is either necessary or sufficient for any given disorder to emerge [3]. The systems exhibit both multifinality (i.e., the same set of causal agents can lead to different mental health outcomes) and equifinality (i.e., diverse causes can produce the same disorder) [4, 5]. The systems are heterogeneous, with individuals differing not only in the specific constellation of symptoms experienced, but also in the etiological factors that precede and predict illness onset [6, 7]. Further, systems within individuals are dynamic: they rise and fall and evolve over time.

In the six articles inaugurating this collection, researchers operating from a range of perspectives review what we know about the systems that affect mental health. In doing so, they chart a path forward for how we can approach the challenging task of better understanding these complex systems, and how we can leverage that understanding to improve treatment.

## Mental health and mental disorder as products of complex systems

McLaughlin et al. [8] provide a review of transdiagnostic risk and resilience factors that mediate the relationship between childhood trauma exposure and psychopathology. In doing so, the authors introduce two ideas that run through much of this collection. First, mental disorders are multifactorial, arising from factors across biological, psychological, and social levels of analysis. McLaughlin and colleagues highlight the role of accelerated biological aging, emotional processing, and social information processing as mediators of the effect of childhood trauma on psychopathology, emphasizing that, by virtue of its impact on these mediators, the same trauma exposure can lead to a range of disorders. Second, the authors emphasize the dynamic nature of mental health and the need to understand how psychopathology unfolds over time, most saliently over the course of childhood and adolescence. McLaughlin and colleagues persuasively argue that interrupting the evolution of these disorders by targeting transdiagnostic factors affected by childhood trauma may be critical to preventing the development of psychopathology.

Ioannidis et al. [9] further explore the effects of childhood maltreatment in an integrated overview of the neurobiological mechanisms underlying resilience. The authors embrace a systems perspective, conceptualizing resilience to adverse events in childhood as a product of a complex biopsychosocial system that includes both bottom-up (genetic and endocrinological) and top-down (social and psychological) influences. These components interact to drive the neurobiological changes observed after childhood maltreatment. Ioannidis and colleagues argue that to conceptualize and to study this system, we should draw from the concepts and methods developed in the interdisciplinary field of complexity science. Illustrating the value of this approach, the authors identify valuable analytic tools from complexity science that can be used to study systems in psychiatric research, and provide a clear conceptual framework for thinking about resilience to childhood adversity as state-space trajectories and attractor states.

Fritz et al. [10] implement one of the suggestions by Ioannidis et al. [9], using network analysis to study interrelations among psychological and environmental resilience factors for mental health, such as family support and self-esteem, in adolescents with and without child adversity, from ages 14 to 17. The authors find that particularly during early adolescence, resilience factors show weaker relations with each other following child adversity, potentially indicating that positive feedback loops among resilience factors (family support → less distress → positive self-esteem → family cohesion → family support) are disrupted. Together with McLaughlin et al. [8], Fritz and colleagues make a compelling case for interventions during childhood and adolescence that target transdiagnostic resilience factors with the aim of preventing the emergence of psychopathology.

The contributions from McLaughlin et al. [8], Ioannidis et al. [9], and Fritz et al. [10] provide an extensive overview of how mental disorders can emerge from the dynamic interactions among components across levels of analysis. Franklin [11] further explores the concept of emergence by shifting the focus to even more elementary components that give rise to psychological phenomena. These components, known as psychological primitives, represent the fundamental and irreducible building blocks of psychological experiences, such as core affect, exteroception, and conceptual knowledge. Drawing heavily from constructivist accounts of emotion, Franklin argues that suicidal thoughts (and all other psychological phenomena) emerge from interactions among these psychological primitives. To understand how distinct biopsychosocial factors contribute to suicidal thoughts, Franklin argues we must understand how they affect these constituent components, a framework that suggests novel paths by which we may intervene to reduce suicidal thoughts.

## Psychological treatment from a complex systems perspective

In the two remaining inaugural contributions of the special collection, Burger et al. [12] as well as Hayes and Andrews [13] leverage a systems-perspective to inform psychotherapy research. Hayes and Andrews [13] provide an introduction of basic principles from complexity science relevant to mental health, such as attractor states (i.e., states to which the mental health system will gravitate), early warning signals that indicate increased potential for a transition to an alternative attractor state, and network destabilization: the disruption of a system stuck in a harmful state to facilitate transitions to a new state of mental health. Across various fields of science, each of these concepts has been fruitfully used to explain, predict, and intervene on systems. In ecology, for example, lakes show early warning signals before they reach a tipping point and transition into an alternative (eutrophic) stable state. Hayes and Andrews review a growing psychiatric literature that draws on these concepts to advance our understanding of psychotherapy and concludes by persuasively arguing that a complex systems approach to psychotherapy is both viable and necessary to transition psychiatry into the twenty-first century.

Burger et al. [12] further embraces a complex systems approach to psychotherapy, calling for the formalization of a patient’s case conceptualization using computational models: a tool commonly used in other areas of complexity science to represent a system and evaluate how that system will evolve over time. Burger and colleagues propose to extend this approach, using computational models as a thinking tool to aid case conceptualizations, a didactic tool for helping patients understand the factors giving rise to their distress, a prediction tool for anticipating treatment outcomes, and a treatment selection tool that can guide the selection of interventions most likely to lead to change in psychotherapy. Together with Hayes and Andrews [13], Burger and colleagues make a compelling argument that a systems approach can inform not only psychiatric research, but also the ongoing practice of psychotherapy.

## Conclusion

A central argument emerges from the six initial articles of this collection: mental disorders arise from complex systems, and our ability to explain, predict, and intervene on mental disorders will hinge on our ability to make progress in understanding these systems. In just a brief sampling from these articles, numerous systems are identified across time scales and levels of analysis, including neurobiological systems, systems composed of psychological primitives that give rise to momentary instances of psychological experience, transdiagnostic systems of risk and resilience factors that evolve throughout adolescence, systems of interacting symptoms that harden into syndromes over months, and social support systems that may evolve over years. These systems overlap, interact, and are embedded within one another: it is systems all the way down. Just as studying one component is insufficient to understand the cause of a mental disorder, studying one system alone is also unlikely to be sufficient. Accordingly, the task of understanding the systems that give rise to mental disorders will be a challenging one.

Yet, with this challenge we bring good news. There is a large and rapidly evolving interdisciplinary body of scientific research dedicated to investigating complex systems [14]. From physics to ecology, scientific efforts have increasingly turned toward unraveling the operation of complex systems. As demonstrated by several articles in this collection, this interdisciplinary work provides a large toolkit from which psychiatry can draw in the effort to understand complex systems in mental health research. This effort calls for us to tear down artificial barriers between disciplines such as psychiatry, clinical psychology, and sociology and highlights the importance of interdisciplinary collaborations. Ultimately, embracing complexity presents both an unparalleled challenge and, simultaneously, an enormous opportunity to advance our understanding of mental disorders and our ability to support those who suffer from them.

## Data Availability

Does not apply.
